# A curated database of rumen ciliate protozoal 18S rRNA gene sequences for metataxonomic applications

**DOI:** 10.1186/s12863-026-01420-y

**Published:** 2026-04-03

**Authors:** Katie Lawther, Ilma Tapio, Arturo Vera-Ponce de León, Velma T. E. Aho, Sharon A. Huws, Nicholas J. Dimonaco

**Affiliations:** 1 Institute for Global Food Security, School of Biological Sciences, Queen’s University Belfast, Belfast, UK; 2 Laboratory of Microbiology, Wageningen University and Research, Wagneningen, Netherlands; 3 Genomics and Breeding, Natural Resources Institute Finland (Luke), Jokioinen, Finland; 4 Faculty of Biosciences, Norwegian University of Life Sciences, Ås, Norway

**Keywords:** Protozoa, Ciliates, 18S, rRNA gene, Rumen, Metataxonomy

## Abstract

**Objectives:**

Protozoa are key members of the rumen microbiome playing significant roles in nutrient cycling and methane production, yet are understudied. As rumen metataxonomic studies increasingly incorporate protozoal primers, the lack of curated dedicated reference databases limits accurate classification. This dataset was developed to address that gap and support future protozoa-focused rumen microbial analyses.

**Data description:**

The curated dataset comprises 228 rumen ciliate protozoal 18S rRNA gene sequences sourced from publicly available datasets. Sequences were processed to remove redundancy and standardise naming. The final database spans 23 families, 53 genera, and 100 species, and is suitable for use in metataxonomic pipelines, including QIIME2. It provides a valuable resource for researchers aiming to improve taxonomic resolution of protozoal communities in rumen environments.

## Objective

The rumen microbiome is a complex and dynamic ecosystem essential to ruminant digestion and health. While bacterial and archaeal constituents have been extensively studied, the eukaryotic members, particularly fungi and protozoa, remain comparatively under-characterised. This disparity stems from challenges with axenic culturing of rumen eukaryotes and the complexity of their genomes [1]. Consequently, metagenomic studies have been heavily biased toward prokaryotic members and metataxonomic approaches have traditionally focused on 16S ribosomal ribonucleic acid (rRNA) gene sequencing.

Despite making up nearly half of the rumen microbial biomass, the biological and metabolic roles of protozoa remain poorly defined in vivo [1, 2]. Defaunation studies, the deliberate removal of protozoa from the rumen, report an increase in microbial protein flow by up to 30% and a reduction of methane emissions by 11%, underscoring their influence on nutrient cycling and greenhouse gas production [2]. Protozoa are major hydrogen producers via their hydrogenosomes, which are specialised organelles that generate $H_2$ during fermentation. This hydrogen production facilitates interspecies transfer to methanogens, with many methanogenic archaea found in close physical association with protozoal cells [3]. While protozoa thus play a central role in methane formation, their impact on methanogen community structure is complex and variable [3].

Considering the importance of protozoa in the rumen microbiome, it is increasingly common to include members of eukaryotic taxa such as ciliate protozoa or anaerobic fungi in rumen metataxonomic studies. However, there is still a lack of appropriate databases specific to protozoal species equivalent to those available for bacterial and archaeal species [4–7]. Therefore, this database was built to support future rumen metataxonomic studies with tools such as QIIME2 [8].

## Data description

These data were sourced from three publications.

1. One hundred and sixty-eight 18S rRNA gene sequences were sourced directly from the supplementary material provided in Kittelmann et al. [9]
2. Sixty-five single amplified genome (SAG) assemblies were sourced from Li et al. [10], PRJNA777442
3. One macronuclear genome of *Entodinium caudatum* was sourced from Park et al. [11], PRJNA380643

Barrnap v0.9 [12] was used with default parameters and –kingdom euk to extract ribosomal RNA genes and those identified as 18S were selected. The other rRNA genes were excluded (28S, 5S_8S and 5S). The 18S rRNA gene sequences from all three sources were then combined (Data file 1 [13]). All sequences were converted to DNA (U-T) and then to remove redundancy in the database, CD-HIT (−est) [14] was used to cluster the sequences at 100% identity and 100% length.

The output from cd-hit-est (.clstr file - Data file 2 [15]), was then manually examined, and for clusters containing multiple identical sequences (length and sequence were identical), only the first sequence in the cluster was kept as a ‘representative’ sequence. The remaining identical sequences were removed and recorded (38 sequences in total, Data file 3 [16]). The sequences that were retained then underwent one of two filtering steps based on their lineages or names.

1. Where IDs (NCBI accession) differed but lineages (provided by the publications) were the same, the sequences were assigned a group number (groups 0–22). The retained *representative* sequence was then renamed to include the allocated group number (all group member information can be found in *Data file 4* [17]).
2. Where the species name differed, the species name was removed and generalised to s__

For example:

**Figure:**
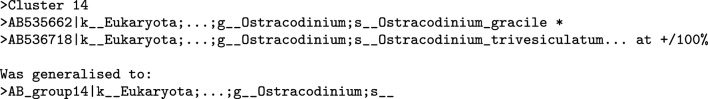

More information and examples regarding the renaming process can be found at the project’s GitHub repository https://github.com/lawkj/Protozoal_18S_rRNA_Database.

This curated database (v1.0.0) contains 228 rumen ciliate protozoal 18S rRNA gene sequences, representing 23 families, 53 genera, and 100 species (RNA sequences Data file 5 [18] and DNA sequences Data file 6 [19], taxonomy Data file 7 [20]).

In addition to the curated 18S rRNA gene sequences, we provide several supporting files formatted for direct use with QIIME2 [8], which were produced using QIIME2 v2024.10.1 with sklearn v1.4.2. Taxonomy information is available both as a plain text file (Data file 7 [20]), and as a QIIME2 ‘artifact’ file (Data file 8 [21]), while the full set of 18S rRNA gene sequences, provided in DNA format, is also included as a QIIME2 ‘artifact’ file (Data file 9 [22]). We also provide a pre-trained Naive Bayes classifier [23] compatible with QIIME2 (Data file 10 [24]) and a QIIME2-formatted BLAST database (Data file 11 [25]). The FASTA sequence files (Data files 5 and 6 [18, 19]) and tab-delimited taxonomy file (Data file 7 [20]) follow standard formats compatible with mothur [26] and other sequence search and classification tools.

Together, this range of provided files enables straightforward integration of the RumenProtozoaDB into QIIME2, mothur and other 18S rRNA gene sequence analysis workflows. Please see Table 1 for full details and links to the data.

**Table 1:** Overview of data files

Label	Data file	File type	Data repository
Data file 1	RumenProtozoaDBv1.0.0.18S.rawsequences	fasta	Zenodo (10.5281/zenodo.18324737) [13]
Data file 2	RumenProtozoaDBv1.0.0.18S.clsr	xlsx	Zenodo (10.5281/zenodo.18324737) [15]
Data file 3	RumenProtozoaDBv1.0.0.18S.filtered prerename	fasta	Zenodo (10.5281/zenodo.18324737) [16]
Data file 4	RumenProtozoaDBv1.0.0.18S.group info	csv	Zenodo (10.5281/zenodo.18324737) [17]
Data file 5	RumenProtozoaDBv1.0.0.18S.RNAsequences	fasta	Zenodo (10.5281/zenodo.18324737) [18]
Data file 6	RumenProtozoaDBv1.0.0.18S.DNAsequences	fasta	Zenodo (10.5281/zenodo.18324737) [19]
Data file 7	RumenProtozoaDBv1.0.0.18S.taxonomy	txt	Zenodo (10.5281/zenodo.18324737) [20]
Data file 8	2024.10.RumenProtozoaDBv1.0.0.18S.taxonomy	qza	Zenodo (10.5281/zenodo.18324737) [21]
Data file 9	2024.10.RumenProtozoaDBv1.0.0.18S.sequences	qza	Zenodo (10.5281/zenodo.18324737) [22]
Data file 10	2024.10.RumenProtozoaDBv1.0.0.18S.nb.sklearn-1.4.2.qza	qza	Zenodo (10.5281/zenodo.18324737) [24]
Data file 11	2024.10.RumenProtozoaDBv1.0.0.18S.blastdb.qza	qza	Zenodo (10.5281/zenodo.18324737) [25]

## Limitations

These data are limited in size, drawing from only three independent studies; however, this reflects the totality of publicly available data for rumen-associated protozoal 18S rRNA gene sequences and genomes. A limitation of this database is that it focuses exclusively on the 18S rRNA region and does not cover alternative regions, such as ITS or 28S, which may also be used for protozoal taxonomic classification. Should additional data become publicly available, the authors intend to expand the database and update the GitHub repository accordingly.

## Data Availability

All data have been made available at https://github.com/lawkj/Protozoal_18S_rRNA_Database, Zenodo 10.5281/zenodo.18324737.

## Abbreviations

rRNA: Ribosomal ribonucleic acid
SAG: Single amplified genome
